# Monoclonal Antibodies that Inhibit the Proteolytic Activity of Botulinum Neurotoxin Serotype/B

**DOI:** 10.3390/toxins7093405

**Published:** 2015-08-26

**Authors:** Yongfeng Fan, Jianbo Dong, Jianlong Lou, Weihua Wen, Fraser Conrad, Isin N. Geren, Consuelo Garcia-Rodriguez, Theresa J. Smith, Leonard A. Smith, Mengfei Ho, Melissa Pires-Alves, Brenda A. Wilson, James D. Marks

**Affiliations:** 1 Department of Anesthesia and Perioperative Care, University of California, San Francisco, San Francisco General Hospital, Room 3C-38, 1001 Potrero Avenue, San Francisco, CA 94110, USA; E-Mails: frank.fan@ucsf.edu (Y.F.); jianbo.dong@ucsf.edu (J.D.); jianlong.lou@ucsf.edu (J.L.); wei.wen@ucsf.edu (W.W.); Conrad.fraser@ucsf.edu (F.C.); MariaConsuelo.Garcia@ucsf.edu (C.G.-R.); 2 Molecular and Translational Sciences Division, United States Army Medical Institute of Infectious Diseases, Fort Detrick, MD 21702, USA; E-Mail: theresa.j.smith.civ@mail.mil; 3 Medical Countermeasures Technology, U.S. Army Medical Research Institute of Infectious Diseases, Fort Detrick, MD 21702-5011, USA; E-Mail: Leonard.A.Smith1.civ@mail.mil; 4 Department of Microbiology, University of Illinois at Urbana-Champaign, Urbana, IL 61801, USA; E-Mails: mho1@illinois.edu (M.H.); melalves@illinois.edu (M.P.-A.); bawilson@life.illinois.edu (B.A.W.)

**Keywords:** botulinum antitoxin, inhibitory antibodies, Botulinum neurotoxin serotype B, alpha-exosite

## Abstract

Existing antibodies (Abs) used to treat botulism cannot enter the cytosol of neurons and bind to botulinum neurotoxin (BoNT) at its site of action, and thus cannot reverse paralysis. However, Abs targeting the proteolytic domain of the toxin could inhibit the proteolytic activity of the toxin intracellularly and potentially reverse intoxication, if they could be delivered intracellularly. As such, antibodies that neutralize toxin activity could serve as potent inhibitory cargos for therapeutic antitoxins against botulism. BoNT serotype B (BoNT/B) contains a zinc endopeptidase light chain (LC) domain that cleaves synaoptobrevin-2, a SNARE protein responsible for vesicle fusion and acetylcholine vesicle release. To generate monoclonal Abs (mAbs) that could reverse paralysis, we targeted the protease domain for Ab generation. Single-chain variable fragment (scFv) libraries from immunized mice or humans were displayed on yeast, and 19 unique BoNT/B LC-specific mAbs isolated by fluorescence-activated cell sorting (FACS). The equilibrium dissociation constants (K_D_) of these mAbs for BoNT/B LC ranged from 0.24 nM to 14.3 nM (mean K_D_ 3.27 nM). Eleven mAbs inhibited BoNT/B LC proteolytic activity. The fine epitopes of selected mAbs were identified by alanine-scanning mutagenesis, revealing that inhibitory mAbs bound near the active site, substrate-binding site or the extended substrate-binding site. The results provide mAbs that could prove useful for intracellular reversal of paralysis and identify epitopes that could be targeted by small molecules inhibitors.

## 1. Introduction

Botulinum neurotoxins (BoNTs), produced by the bacterium *Clostridium botulinum* are the most lethal substances known [1] and are considered to be a high risk for bioterrorism use [2]. All of the serotypes of BoNTs are composed of two polypeptide chains and three functional protein domains [3]. The 100-kDa heavy chain (H_C_) contains the binding domain (H_C_) and translocation domain (H_N_) and the 50-kDa light chain (LC) contains the zinc protease catalytic domain. The C-terminal domain of the HC (H_C_) binds receptors on the presynaptic membrane [4,5,6,7,8,9] leading to BoNT endocytosis. In the neuron, the N-terminal domain of the H_C_ (H_N_) forms a channel across the endosomal membrane allowing delivery of the LC into the cytoplasm [10,11]. In the case of BoNT/B, the protease cleaves synaptobrevin-2 (Syb-2), a SNARE protein, resulting in loss of neurotransmitter release and flaccid paralysis (botulism) [12]. BoNTs have stringent specificity requirements and low turnover due to their extended substrate-binding sites [13]. In the holotoxin, the H_N_ “belt” wraps around the catalytic domain and occludes the extended substrate-binding site. The protease is inactive until the H_N_ and belt separate from the LC during the translocation process inside the neuron [3,14].

The only approved treatment for botulism is human or equine polyclonal antitoxin antibodies used to treat infant and adult botulism, respectively [15,16]. To replace equine antitoxin, we have generated a number of extremely high-affinity recombinant monoclonal antibodies (mAbs) to BoNTs [17,18,19] that neutralize the toxins by a variety of mechanisms, including clearing BoNT from the circulation before it can reach the neuron or preventing BoNT entry into neurons [17]. Such recombinant antitoxins for serotypes A, B, C, D and E are in clinical or pre-clinical development [20,21]. Antibodies and antitoxins, however, cannot reverse BoNT paralysis, as they do not cross the neuronal cell membrane. An alternative to antitoxins is small molecule inhibitors of the catalytic domain [22,23,24]. Small molecule inhibitors are at a very early stage of research development; none have been approved for treatment and none have advanced into pre-clinical or clinical development. Obstacles hindering advancement of antitoxin therapies include the difficulty in development of potent inhibitors with exquisite specificity and high affinity and the challenges of getting them selectively into the presynaptic neuron [22,23,25].

Alternatively, BoNT antibodies could potentially inhibit translocation or proteolysis if they could be taken up into the neuron and then also delivered into the cytosol of the neuron via attachment to the toxin. A number of platforms are currently being developed for targeted delivery of therapeutic cargos, recently reviewed in [26]. The advent of these new post-exposure strategies potentially enables the delivery of antibody-based therapies to the site of toxin action in neurons, as has been reported for the delivery of inhibitory peptides [27].

We previously reported the isolation of a single-domain camelid VHH antibody that bound the BoNT/A LC alpha exosite with a K_D_ of 147 pM and potently inhibited SNAP25 cleavage [28]. More recently we have reported scFv and IgG mAbs that bind BoNT/A LC and inhibit SNAP25 cleavage, and like the VHH, these inhibitory mAbs bind at the alpha exosite [29]. Here, we report generation of mouse and fully human antibodies that can inhibit BoNT/B LC proteolytic activity, as well as identification of the mAb epitopes mediating this inhibition.

## 2. Results and Discussion

### 2.1. Libraries Used for Monoclonal Antibody Generation

To generate mAbs that bind BoNT/B LC, yeast display scFv antibody libraries were constructed from immunized humans and mice. Humans were immunized with pentavalent (ABCDE) toxoid and mice were immunized with one of the BoNT/B sub-serotypes or recombinant BoNT/B LC (Table 1), using the immunization strategy described in the methods. scFv yeast display libraries were constructed from antibody variable (V) region genes isolated from either peripheral blood lymphocytes (human libraries) or from spleens (murine libraries). Yeast display libraries were flow sorted for binding to either BoNT/B or recombinant BoNT/B LC. After three to four rounds of sorting, a total of 19 unique scFvs, as determined by DNA sequencing, were identified that bound BoNT/B LC (Table 1).

**Table 1:** Libraries used for BoNT/B LC mAb generation.

Library	Immunizing Antigen	Vector	Library Size	mAbs
Human donor libraries	BoNT/A-E Toxoid	pYD2	>10^7^	1B10.1, 1B22, 2B23, 2B25.1, 4B19, B6.1
Mouse B1 *	BoNT/B1 Okra	pYD4	4.0 × 10^7^	16B3, 18A6, 18D10, 18E5, 18F2, 19A9, 19D22, 19G6
Mouse B LC	BoNT/B1 LC	pYD4	5.0 × 10^7^	31A5, 31E2, 31G2, 31H3, 34E8

* Mice were immunized with BoNT/B H_C_ fragment then boosted with the indicated holotoxin.

### 2.2. Characterization of Monoclonal Antibodies 

**Figure 1:**
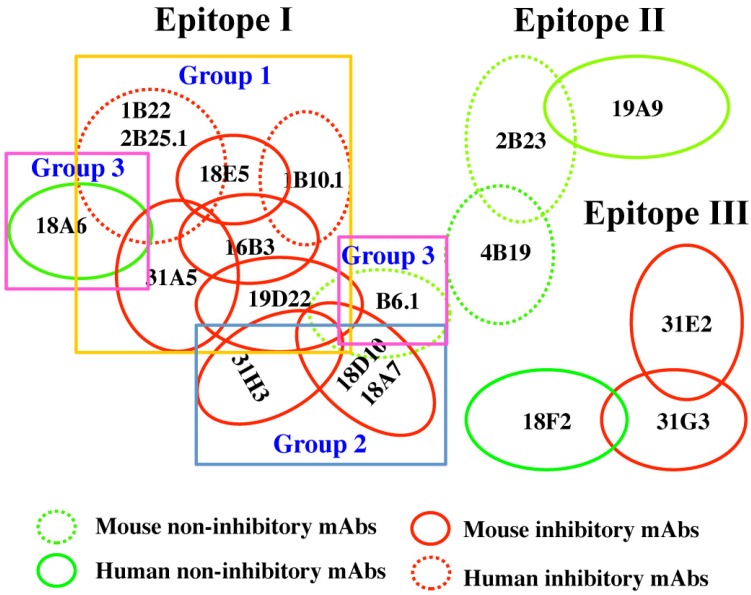
Cartoon of mAb epitope clusters. mAbs were clustered based on their ability to simultaneously bind BoNT/B LC with one of the scFv displayed on the surface of yeast used to capture BoNT/B LC out of solution and then the ability of a second scFv to bind the captured B LC determined. mAb epitopes are shown as circles; overlapping circles indicate mAb pairs that cannot simultaneously bind LC. Red circles indicate mAbs that inhibited BoNT/ B endopeptidase activity; green circles represent non-inhibitory mAbs. Dotted circles indicate human-derived mAbs, and solid circles mouse-derived mAbs. Epitope cluster 1 antibodies were sub-divided into three groups based on degree of inhibition of BoNT/ B endopeptidase activity.

Nineteen mAbs with unique VH CDR3 binding BoNT/B LC were isolated from the human and mouse libraries, as determined by DNA sequencing (Table 1 and Table 2). Equilibrium binding constants (K_D_) for yeast-displayed scFv binding to BoNT/B LC ranged from 0.24–14.3 nM with an average K_D_ of 3.27 nM (Table 2). The epitopes recognized by each scFv were classified into epitope groups based on their ability to compete with each other for binding to BoNT/B LC. In the assay, BoNT/B1 LC (or holotoxins of other subserotypes) captured by yeast-displayed scFv was probed with *E. coli*-expressed soluble scFv. The 19 mAbs were grouped into three epitope clusters (I-III) based on their ability to compete for LC binding (Figure 1). The largest cluster (I) was shared by 13 scFvs, at least one of each overlapped with other cluster I mAbs. Given the large number of mAbs, the epitope cluster was further divided into three subgroups (I-1, I-2 and I-3) based on the degree of inhibition. 

**Table 2:** Equilibrium binding constants (K_D_) of BoNT/B LC binding mAbs isolated from mouse and human scFv libraries. K_D_ values were measured on the yeast-displayed scFv using soluble BoNT/B LC by flow cytometry. Cross reactivity was determined by measuring the K_D_ values for the different holotoxin subserotype.

scFv	mAb Origin	Epitope-Group	Affinity K_D_ × 10^−9^ M (± SD)	Subserotype cross Reactivity			
			B LC	B1	B2	B3	B4
B6.1	Human	I-3	0.24 (±0.14)	++++	+++	+++	+++
1B10.1	Human	I-1	14.33 (±3.73)	++	++	+	+++
1B22	Human	I-1	1.05 (±0.38)	++++	++++	++++	++++
2B23	Human	II	0.60 (±0.22)	++++	++++	++++	++++
2B25.1	Human	I-1	0.24 (±0.13)	++++	++++	++++	++++
4B19	Human	II	0.54 (±0.23)	++++	+++	+++	+++
16B3	Mouse	I-1	0.84 (±0.22)	++++	++++	++++	++++
18A6	Mouse	I-3	4.21 (±0.36)	+++	++	++	++
18A7	Mouse	I-2	13.5 (±3.02)	+	+	++	+
18D10	Mouse	I-2	7.53 (±1.72)	+++	+++	+++	+++
18E5	Mouse	I-1	1.00 (±0.17)	+++	+++	+++	+++
18F2	Mouse	III	1.19 (±0.16)	+	+++	++	-
19A9	Mouse	II	3.16 (±0.17)	+++	++++	+++	++++
19D22	Mouse	I-1	9.31 (±1.98)	+++	+++	+++	+++
31A5	Mouse	I-1	1.75 (±0.13)	-	-	-	-
31E2	Mouse	III	17.0 (±2.46)	-	-	-	-
34E8	Mouse	I-2	1.08 (±0.17)	-	-	-	-
31G2	Mouse	III	3.34 (±0.37)	-	-	-	-
31H3	Mouse	I-2	1.39 (±0.34)	+++	+++	+++	+++

++++ indicates K_D_ value on yeast <1.0 nM; +++ indicates 1.0–10.0 nM; ++ indicates 10–50 nM; + indicates >50 nM; - indicates that mAbs did not bind holotoxin.

### 2.3. Inhibition of BoNT LC Endopeptidase Activity

**Figure 2:**
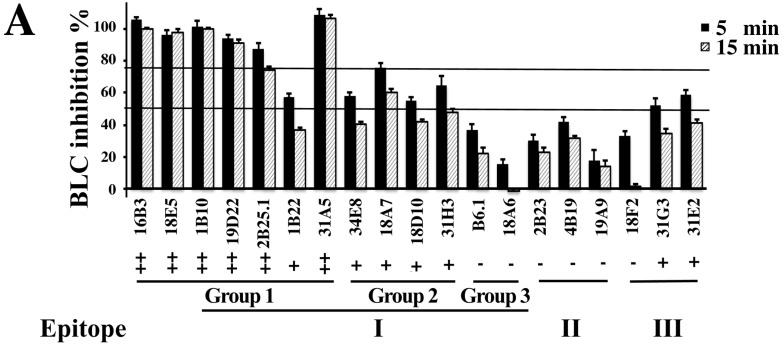

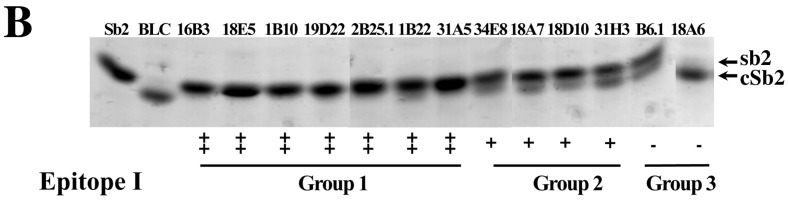
Inhibition of BoNT/B LC endopeptidase activity by mAbs. (**A**) FRET-based screening assay for mAb inhibitors of BoNT/B LC using FRET. The ratio of Fluorescence at 528/485 nm (R) is used for evaluation of BoNT/B LC-mediated YsCsY cleavage. The inhibition of BoNT/B LC activity (%) was calculated as (R_mAb_ − R_BLC_)/(YsCsY − R_BLC_) × 100%. Extent of inhibition is denoted as “++”, ≥75% inhibition at 5 min.; “+”, ≥50% inhibition at 5 min; “−”, ≤50% inhibition at 5 min; (**B**) BoNT/B LC inhibition assayed by SDS-PAGE. Synaptobrevin-2 (Syb-2) and mAbs were mixed together in Tris buffer and BoNT/B LC (20 nM) was added to start the reaction. After 15 min, protein-loading buffer was applied to stop the reaction. Cleaved Syb-2 is indicated as cSb2. Extent of inhibition is denoted as “++”, ≥75% inhibition; “+”, ≥50% inhibition; “−”, ≤50% inhibition at 15 min. The experiments were performed in triplicate. The mean ± SD is presented.

A Fluorescence Resonance Energy Transfer (FRET) assay was used screen each scFv for inhibition of substrate cleavage by BoNT/B LC. mAbs in epitope group 1, cluster I showed the greatest inhibition with scFvs 18E5 and 1B10.1 completely inhibiting substrate cleavage after 30 min. of incubation, while scFvs 16B3, 19D22 and 31A5 decreased the cleavage rate by 11–40 fold (Figure 2A). The remaining scFvs in epitope cluster I group 1 (2B25.1 and 1B22) showed less than a 4.5-fold reduction in the cleavage rate, similar to that seen for scFvs in epitope group 1, cluster II. scFv in epitope cluster I, group 3, as well as mAbs in epitope clusters II, and III showed minimal (at most 1.63-fold) inhibition. The degree of inhibition of substrate cleavage did not correlate with affinity of the scFv for BoNT/B LC. SDS-PAGE was also used to independently verify the ability of scFvs in epitope group I to inhibit substrate (Syb-2) cleavage. SDS-PAGE results were generally but not entirely consistent with inhibition of proteolytic activity, as determined using FRET (Figure 2B).

IC_50_ values of mAbs displaying the most potent inhibition were measured using FRET (Table 3). IC_50_ values ranged from 2 nM to 59 nM, with the lowest values for those mAbs (1B10.1, 16B3, 18E5 and 19D22) that showed the greatest slowing in the substrate cleavage rate. We are unable to explain how 1B10.1 had an IC_50_ less than the KD, however these were two different assay types perform in different buffers. Of note, the 1B10.1 IC_50_ was consistent with the amount of cleavage observed in Figure 2.

**Table 3:** IC_50_ values of selected mAbs.

mAb	Format	IC_50_ (×10^−9^ M) ^a^	K_D_ for BoNT/B1LC on Yeast (×10^−9^M) ^b^	Epitope Group
1B10.1	IgG	2.09 (2.04–2.14)	118 ^b^ (69.0–197)	1
16B3	scFv	5.21 (3.04–7.39)	0.84 (0.40–1.28)	1
18E5	scFv	7.01 (6.09–7.93)	1.00 (0.66–1.34)	1
19D22	scFv	5.47 (2.48–8.46)	9.31 (5.35–13.3)	1
18D10	scFv	59.0 (54.8–63.3)	7.53 (4.09–11.0)	2

^a^ Measured using FRET assay. 95% confidence intervals are given; ^b^ The K_D_ of 1B10.1 IgG was measured by flow fluorimetry in a KinExA. All other K_D_ values were measured on yeast-displayed scFv. 95% confidence intervals are given.

### 2.4. Defining the mAb Epitopes

**Figure 3:**
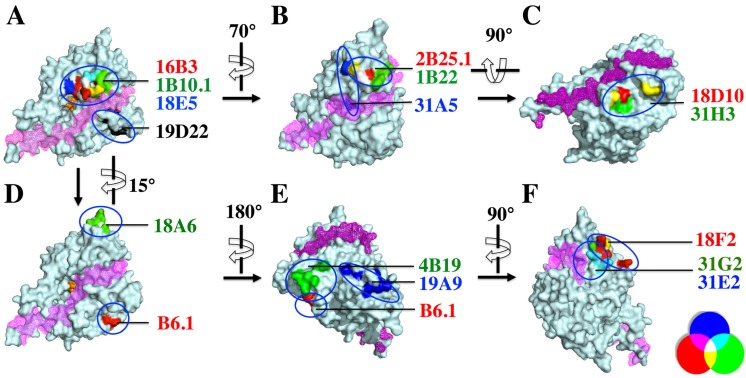
Fine epitopes of BoNT/B LC-binding mAbs. For each mAb, amino acids that result in loss of mAb binding are modeled on the crystal structure of BoNT/B LC (pdb ID: 1S0F). The BoNT/B LC is shown in cyan and the belt-binding groove in pink mesh. Structures B through F were generated by rotation of the BoNT/B LC strucutre in panel A as illustrated by the curved arrows. (**A**) The BoNT/B catalytic site is shown as an orange sphere. In each panel (**A**–**F**) the mAb name is colored to reflect the color of the corresponding residue in the epitope. Where a residue is shared by more than one mAb, the amino acid color is changed to reflect overlap (see legend, lower right). Panels (**A**,**B**) show the epitopes of mAbs in group 1 of epitope I; panel (**C**), mAbs in epitope cluster I, group 2; panel (**D**), mAbs in epitope cluster I, group 3; panel (**E**), mAbs in epitope cluster II; and panel (**F**), mAbs in epitope cluster III.

The fine epitopes of 17 of the mAbs were determined using yeast display [30], in order to define the binding sites on the BoNT/B LC that resulted in inhibition of endopeptidase activity. Mutations were randomly introduced into the BoNT/B LC gene using error prone PCR, and the resulting mutants were displayed on the surface of yeast. Each of the 19 mAbs was incubated separately with the yeast displayed BoNT/B LC library, and the yeast cells were sorted for loss of mAb binding. After three rounds of sorting with decreasing antigen concentration, individual yeast cells were analyzed to identify those not binding mAbs, and the BoNT/B LC gene was sequenced to identify the mutations responsible for loss of binding (Table 4). These mutations were then mapped onto the crystal structure of BoNT/B LC [31]. While a structure of substrate-bound BoNT/B LC has not been reported, one can use the BoNT/B belt as a substrate surrogate [32]. The belt-binding site is shown in pink in Figure 3. One of the mAbs in epitope cluster I, group 2 (34E8) could not be mapped due to limited expression of soluble scFv/IgG.

**Table 4:** BoNT/B LC mutants that result in loss of mAb binding. mAbs are grouped (separated by a solid line) based on shared amino acids. Amino acids indicated in bold were shared by the epitopes of more than one mAb.

Epitope	mAb	Amino Acids Affecting mAb Binding
Epitope cluster I group 1	16B3	**G119,** D120, **Q177**, N178, **H179**, R183, G185, I189
	18E5	Y117, **G119**, **R121**, R122, I132, A133, **Q177**, **H179**
	1B10.1	R121, H179, D244
	2B25.1	Y117, G119, E127, C308, **I309**
	1B22	E127, P312, **I309**, I314, N315
	31A5	G188, G185, P124, V135, H236, A133, **I309**
	19D22	E22, A26, R31, P55
Epitope cluster I group 2	18A7	R31, P55, T501
	18D10	R31, P55, T501
	31H3	**R31**, R48, **P55**, F58
Epitope cluster I group3	B6.1	N81, K84
	18A6	N303, L306, V307, K320, D332, E334, K336, S338
Epitope cluster II	4B19	N6, N8, N10, D11, P12
	19A9	T392, I393, N396, S400, K402, D403, R409, Q411, V416
Epitope cluster III	18F2	K285, **V341**, E342K, D345, L347
	31G2	**V341**, S343, **K346**, K349
	31E2	L238, F295, K326, G237, **K346**, **K349**

**Figure 4:**
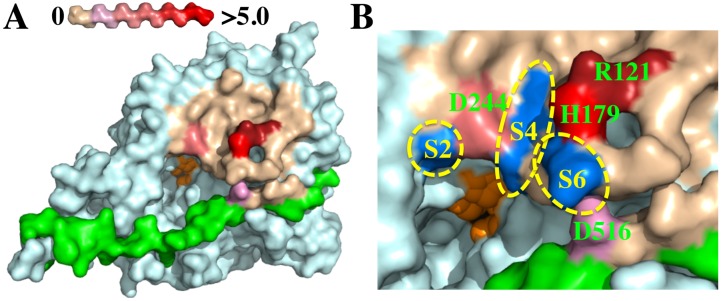
Binding epitope of mAb 1B10.1 on BoNT/B surface. Shown are molecular models constructed with Pymol software based on the BoNT/B crystal structure (pdb ID: 1S0F). (**A**) Holotoxin LC structure with color-coding indicating the change in ΔΔG (kcal/mole) of binding of 1B10.1 using the scale shown at top left. The residues comprising the active site are shown in orange; (**B**) Expanded view of the 1B10.1 epitope on the surface of BoNT/B with color-coding as in (A). Substrate-binding S-pockets are shown in blue. The 1B10.1 epitope includes the S2 amino acid D244 and would cover the S4 and S6 binding sites of Syb-2.

Epitope location determined by the above method (Figure 4) correlated well with the classification of epitopes based on ability of mAbs to bind simultaneously to BoNT/B LC (Figure 1). The three mAbs displaying the most potent inhibition (16B3, 18E5 and 1B10.1, epitope cluster I, group 1) bound an epitope located near the catalytic site and the putative substrate-binding site and shared amino acids in the epitope (Figure 3A). The mAb 19D22, displaying the next most potent inhibition, also bound near the catalytic and substrate-binding sites, but at a structurally non-overlapping epitope with mAbs in the group 1 below the belt (Figure 3A). The remaining inhibitory mAbs in epitope cluster I, group 1 (2B25.1, 1B22 and 31A5) bound above and near the substrate-binding site but remote from the catalytic site (Figure 3B). The epitope of 31A5 includes an amino acid within the substrate-binding site, explaining why it is more inhibitory than 2B25.1, 1B22. In epitope cluster I, group 2, the weakly inhibitory mAbs 18A7, 18D10 and 31H3 shared amino acids in the epitope and bound an epitope below but near the substrate-binding site and more remote from the catalytic binding site than mAbs in epitope cluster I, group 1 (Figure 3C). In epitope cluster I, group 3, the non-inhibitory mAbs B6.1 and 18A6 bound far from the substrate-binding site (Figure 4D). Similarly, in epitope cluster II (mAbs 4B19, 19A9) and III (mAbs 18F2, 31G2 and 31E2), the minimally inhibitory or non-inhibitory mAbs bind remote from the substrate-binding site. The antibodies binding in these epitopes that displayed low levels of inhibition may be conformationally-specific, stabilizing catalytically inactive form of the enzyme [14]. Conformationally-specific antibodies have been identified for other proteases [33]. Residue K285 of the epitope of 18F2 is adjacent to one of the reported Ca^2+^-binding sites that includes R284 [34].

### 2.5. The Epitope of the Inhibitory mAb 1B10.1 Includes Active Site Residues 

The fine epitope of the highly inhibitory mAb 1B10.1 was further mapped by measuring the loss of free energy of binding (ΔΔG) that occurred when the side chain of amino acids in the epitope were exchanged for alanine [18,30] (Table 5). Amino acids around the 1B10.1 binding site were individually mutated to alanine and displayed on yeast, and their K_D_ values *vs.* F(ab) 1B10.1 were measured to calculate ΔΔG values. Four amino acids (indicated in bold in Table 5) were involved in the 1B10.1 epitope. Three are on the LC (R121, H179, D244) and one is on the H_N_ belt (D516). As shown in Figure 4A, mAb 1B10.1 binds at the active site (shown in orange) and overlaps with the S2, S4 and S6 binding sites for Syb-2 (Figure 4B). That one of the critical amino acids for 1B10 binding (D516) is on the belt explains the observation that 1B10.1 binds to the BoNT/B holotoxin with much higher affinity than to the catalytic domain alone (Figure 4B, Table 2). 

**Table 5:** Equilibrium binding constants (K_D_) and loss of free energy of binding (ΔΔG) to 1B10.1 upon mutation of the indicated BoNT/B LC amino acid to alanine. Amino acids listed in bold indicate those critical for binding.

Mutation of BoNT/B1 LC	Mean K_D_^a^ (±SD)	Ratio Mutant/Wild Type	ΔΔG
**R121A**	No binding - ≥5.0	-	≥5.0
**R122A**	89.57 (±2.31)	0.54	−0.36
**V123A**	106.25 (±4.45)	0.64	−0.26
**P124A**	105.90 (±8.06)	0.64	−0.26
**L125A**	105.95 (±1.20)	0.64	−0.26
**I132A**	99.38 (±2.86)	0.60	−0.30
**Q177A**	127.10 (±6.51)	0.76	−0.16
**N178A**	110.55 (±9.12)	0.66	−0.24
**H179A**	**14621.50 (±3178)**	**87.92**	**2.61**
**F180A**	90.55 (±15.63)	0.54	−0.35
**R183A**	100.25 (±23.8)	0.60	−0.29
**E184A**	89.29 (±52.3)	0.54	−0.36
**F186A**	97.24 (±25.4)	0.58	−0.31	
**V243A**	99.78 (±18.3)	0.60	−0.30
**D244A**	**1349.37 (±523)**	**8.11**	**1.22**
**D245A**	123.65 (±20.7)	0.74	−0.17
**Q264A**	127.75 (±21.6)	0.77	−0.15
**K290A**	154.20 (±74.8)	0.93	−0.04
**Q293A**	114.88 (±22.1)	0.69	−0.22
**N294A**	126.05 (±26.8)	0.76	−0.16
**G297A**	127.70 (± 34.2)	0.77	−0.15
**I298A**	97.71 (±11.7)	0.59	−0.31
**R301A**	93.56 (±20.4)	0.56	−0.33
**D512A**	119.30 (±50.9)	0.72	−0.19
**N514A**	145.10 (±26.6)	0.87	−0.08
**V515A**	115.80 (±20.1)	0.70	−0.21
**D516A**	**274.90 (±42.8)**	**1.65**	**0.29**
**Wild Type**	166.30 (±84.7)	-	-

^a^ Mean of three measurements.

We previously reported that the antibodies that most potently inhibited BoNT/A are bound at the alpha-exosite, where the α-helix of substrate SNAP-25 binds, and are remote from the substrate cleavage site [28,29]. In contrast, the mAbs showing the greatest inhibition of BoNT/B activity bound at the catalytic site with the degree of inhibition decreasing with distance of the epitope from the catalytic site. This is consistent with data indicating that truncated Syb-2 with as few as 21 residues upstream of the cleavage site are required for high-affinity binding and cleavage of Syb-2 [35,36]. In contrast, SNAP-25 binding and cleavage is significantly reduced when the SNAP-25 N-terminal helix that binds to the alpha-exosite is truncated [14]. 

## 3. Experimental Section

### 3.1. Ethics Section

The USAMRIID Institutional Animal Care and Use Committee approved the animal care and use protocol to conduct the animal studies reported here. Research was conducted under an IACUC approved protocol in compliance with the Animal Welfare Act, PHS Policy, and other Federal statutes and regulations relating to animals and experiments involving animals. The facility where this research was conducted is accredited by the Association for Assessment and Accreditation of Laboratory Animal Care, International (AAALAC/I) and adheres to principles stated in the Guide for the Care and Use of Laboratory Animals, National Research Council, 2011. The specific national regulations and guidelines to which this animal care and use protocol adheres are the following: (1) 7 United States Code, Sections 2131-2159, Chapter 54 “Animal Welfare Act”, and (Code of Federal Regulations, Chapter 1, Subchapter A, Parts 1-4 “Animal Welfare Regulations”; (2) Health Research Extension Act of 1985, Public Law 99-158 “Animals in Research” and the Public Health Service Policy in Humane Care and Use of Laboratory Animals; (3) Biosafety in Microbiological and Biomedical Laboratories, 5th Edition, National Institute of Health, Human and Health Services Publication (CDC) 21-112; (4) Army Regulation 40-33 “The Care and Use of Animals in DOD Research, Development, Test and Evaluation or Training Programs” and (5) DOD Instruction 3216.01 “Use of Animals in DOD Programs”. USAMRIID is accredited by AAALAC/I. This agency uses the publication titled “The Guide for the Care and Use of Laboratory Animals”, 8th Edition, Institute for Laboratory Animal Research, National Research Council, as a guideline for evaluation and accreditation of program and it is based on the actual national regulations and guidelines for animal care and use programs. The animals used in this study were euthanized using carbon dioxide gas following the AVMA Guidelines on Euthanasia prior to spleen removal. 

The University of California, San Francisco (UCSF) Institutional Review Board approved the human use protocol used for the studies described here. Human donors were laboratory workers being immunized to work with BoNT who were recruited via an informational letter and who signed informed consent under a protocol approved by the UCSF IRB.

### 3.2. Oligonucleotides for Library Construction

The primers for site-directed mutagenesis were designed and synthesized per the QuikChange^®^ Site-Directed Mutagenesis Kit (Agilent Technologies, Santa Clara, CA, USA) instructions. The primers for human and mouse library construction were synthesized as described previously [29,37]. 

### 3.3. Strains, Media, Antibodies, and Toxin

YPD medium was used for growth of *Saccharomyces cerevisiae* strain EBY100, SD-CAA, for selection of pYD4 transformed EBY100 and SG-CAA, for induction of scFv expression on the surface of EBY100. *E. coli* strain DH5α was used for subcloning and preparation of plasmid DNA. *E. coli* strain BL21 was used for BoNT/B LC fragment and Syb-2 expression. *E. coli* strain TG1 was used for soluble scFv antibody expression. Pure holotoxin BoNT/B1 was purchased from Metabiologics (Madison, WI, USA). Other subserotypes were obtained from U.S. Army Medical Research Institute of Infectious Diseases, and from Dr. Eric Johnson, University of Wisconsin. All IgGs were expressed from Chinese hamster ovary (CHO) cells, while the mouse anti-SV5 antibody was purified from hybridoma cells, and labeled with AlexaFluo-488 or AlexaFluo-647 labeling kit (Invitrogen, Carlsbad, CA, USA). All the secondary antibodies, including PE or AlexaFluo-647-conjugated goat anti human-Fc, goat anti-mouse Fc and goat anti-human F(ab) (Jackson ImmunoResearch Laboratories, West Grove, PA, USA).

### 3.4. Protein Expression and Purification

The BoNT/B LC (amino acid residues 1–441) encoding cDNA was amplified from a synthetic BoNT/B1 gene. The Syb-2-encoding cDNA was purchased from ATCC (MGC-45137). The cDNAs were subcloned into the plasmid pET15b and then the protein was expressed in *E. coli* BL21 (DE3) cells induced by 0.5 mM IPTG at 18 °C overnight. The cells were broken with Novagen BugBuster^®^ Master (EMD Millipore, Billerica, MA, USA), and hexahistidine-tagged BoNT/B LC was purified by immobilized metal affinity chromatography (IMAC) using Ni-NTA agarose (Qiagen, Valencia, CA, USA) followed by cation exchange chromatography.

For scFv expression, the scFv genes of the selected antibodies that bound to BoNT/B LC were subcloned into the expression vector pSYN1 [38] and were transformed into *E. coli* TG1 cells. Following induction with 0.5 mM IPTG at 18 °C overnight, periplasmic proteins were extracted by osmotic shock and the hexahistidine-tagged scFvs were purified by IMAC using Ni-NTA agarose. 

The plasmid for expression of the BoNT/B-specific Syb-2-derived FRET-substrate, yellow fluorescent protein-synaptobevin-2-cyan fluorescent protein-synaptobevin-2-yellow fluorescent protein (YsCsY) with a hexahistidine affinity tag at the N-terminus and 16 repeats of Asp-Glu (DE-tag) at the C-terminus was constructed as described previously for the BoNT/A-specific SNAP25-derived FRET substrate [39,40], except with residues 27–94 of mouse Syb-2 as the substrate peptide sequence. YsCsY was purified from cell-free extracts of *E coli* BL21 cells after IMAC using Ni-NTA agarose, followed by anion-exchange chromatography.

### 3.5. Mouse Immunization and Spleen Harvest

Sixteen female CD-1 mice were vaccinated three times at four-week intervals with 5 µg of BoNT/B-HC to establish protection against potentially fatal active toxin challenges. Immunized mice were then challenged with 20,000–200,000 LD_50_ of BoNT/B1 (Okra, five mice per group) at 11, 14, and 16 weeks or at 11, 16, and 19 weeks. Four additional mice were immunized with BoNT/B LC as above but without holotoxin challenge. Mice were euthanized and spleens were removed five days after the final toxin challenge/boosts. Studies using mice were conducted in compliance with the Animal Welfare Act and other federal statutes and regulations relating to animals and experiments involving animals, and adhere to principles stated in the Guide for the Care and Use of Laboratory Animals, National Research Council, 2011. The facility where this research was conducted is fully accredited by the AAALAC/I.

### 3.6. Yeast Displayed scFv Library Construction and Library Sorting

Total RNA was isolated from mouse spleens or from white blood cells of 12 adult human donors immunized with BoNT toxoids subtype A, B, C, D and E. The cDNA was synthesized by RT-PCR with oligo dT as the primer using a ThermoScript RT-PCR Kit (Invitrogen, Carlsbad, CA, USA). The V_H_ and V_K_ gene repertoires were amplified from cDNA by PCR with primers without gap tails and then amplified with gap-tailed primers, following gel purification for library construction. For the mouse libraries, V_K_ gene repertoires were first cloned into the BssHII/NotI sites of the plasmid pYD4 leading to a pYD4-V_K_ library [29]; then the gap-tailed V_H_ genes were transformed into EBY100 together with the NcoI/SalI digested pYD4-V_K_ libraries using LiAC as described previously [41]. For the human libraries, the V_H_ and V_K_ genes first were coupled with a (G_4_S)_3_ linker to obtain full-length scFv genes by splicing using overlap extension PCR as previously described [37]. The scFv gene was inserted into the NcoI/Not I sites of pYD2 plasmid and transformed into EBY100. The library size was determined by plating serially diluted transformation mixture on SD-CAA plates. The scFv libraries were induced by culturing in SG-CAA medium with 10% SD-CAA for at least 24 h.

For library sorting, the libraries were incubated with 50 nM of BoNT/B LC at RT for 1 h. All subsequent washing and staining steps were performed at 4 °C using ice-cold FACS buffer (phosphate-buffered saline, 0.5% bovine serum albumin, pH 7.4). Washed yeast clones were incubated with 2 μg/mL of 1B10.1 and B6.1 mAbs for 60 min, washed, and then incubated with 1 μg/mL of PE-labeled goat anti-human Fc antibody (Jackson ImmunoResearch, West Grove, PA, USA) and 1 μg/mL Alexa-647-labelled anti-SV5 mAb. After washing, yeast clones were flow sorted on a FACSAria II, and the population with BoNT/B LC-binding yeast was gated and collected. The collected yeast clones were cultured and induced for the next round of sorting. After three rounds of sorting, the collected yeast clones were plated on SD-CAA medium and cultured at 30 °C for 48 h. Individual colonies were picked, grown, and induced in 96 deep-well plates. These colonies were then screened for binding using the same staining conditions used for sorting. Unique BoNT/B LC-binding clones were identified by DNA sequencing.

### 3.7. Measurement of Yeast Displayed scFv K_D_

The equilibrium dissociation constant (K_D_) of yeast-displayed scFvs was measured by flow cytometry, as previously described with modification [28,41]. Briefly, 1 × 10^6^ yeast displaying scFvs were incubated for 1 h in FACS buffer with six different concentrations of BoNT/B LC or holotoxin that spanned the range 10-fold above and 10-fold below the expected K_D_ at room temperature. Ice-cold FACS buffer was used to wash the samples, and 2 μg/mL each of 1B10.1 and B6.1 was applied at 4 °C for 60 min, followed by 1 μg/uL of PE-conjugated goat anti-human IgG and 1 μg/mL Alexa-647-labelled anti-SV5 mAb at 4 °C for 30 min. Finally, the yeast clones were washed with ice-cold FACS buffer and the mean fluorescence (MFl) of binding was measured by flow cytometry. The MFI was plotted against the concentration of antigen and the K_D_ was determined by the following equation:

y = m_1_ + m_2_ × m_0_/(m_3_ + m_0_)

where y = MFI at a given antigen concentration, m_0_ = Antigen concentration, m_1_ = MFI of the no antigen control, m_2_ = MFl at saturation, and m_3_ = K_D_. 

### 3.8. Classification of mAbs Based on Overlap of Epitopes

mAbs were classified into epitope groups based on their ability to compete with each other for binding to antigen. Briefly, yeast-displayed scFvs were incubated for 60 min with 25 nM of BoNT/B LC in solution, and then 50 nM of soluble myc-tagged scFv mAb was added and incubated at 4 °C for 60 min. The ability of soluble scFv to bind to BoNT/B LC was detected by incubation for 60 min. with 1 μg/mL of PE-conjugated anti-myc antibody and 1 μg/mL of Alex-647-labeled SV5 antibody. Binding was measured by flow cytometry. The soluble scFv that bound an overlapping epitope to yeast displayed scFv showed no PE-signal, while those binding non-overlapping epitopes showed a positive PE signal.

### 3.9. MAb Inhibition of Synaptobrevin-2 Cleavage by BoNT/B LC 

For FRET-based analysis of substrate cleavage, 0.8 μM of the BoNT/B-specific Syb-2-derived FRET substrate, YsCsY, was mixed with 200 nM of scFv or IgG in FRET buffer (20 mM HEPES, pH 7.5, containing 1.25 mM DTT, 10 μM ZnCl_2_, 0.2% Tween20, 0.1 mg/mL BSA) in a black 96-well plate (Corning, New York, NY, USA). After pre-incubation for 30 min, 4 nM BoNT/B LC was added to initiate the reaction. With the excitation at 425 nm and a cutoff at 435 nm, the emission at 528 nm and 485 nm was measured at t = 0 and at 5 min intervals using a fluorescence reader (Spectra Max Gemini, Molecular Devices LLC, Sunnyvale, CA, USA). The ratio of Fluo528/485 (R) is used for evaluation of YsCsY cleavage. The extent of inhibition of BoNT/B activity (%) was calculated as (R_mAb_ − R_BLC_)/(R_YsCsY_ − R_BLC_) × 100%, where R_mab_ is the 528/485 nm ratio of a sample with mAb, R_BLC_ is the ratio of the control using BoNT/B LC only and R_YsCsY_ is the ratio of the sample with YsCsY only. The extent of inhibitory activity of the mAb was denoted as the most potent “++” when activity was greater than 75% at 5 min, as medium potency “+” when, >59%, or as having no inhibitory capability “−” when <50% at 5 min. The reaction rate (*v*, change in the rate of the 528/485 nm ratio) was calculated by fitting *v* over the first 10 min to a simple linear regression model: *Y* = *v_0_X* + C, where *Y* = 528/485 nm, *v_0_* = the initial rate (slope), *X* = time, and C = y-intercept.

For SDS-PAGE analysis of substrate cleavage, 200 nM of BoNT/B LC and 8 μM of scFv were mixed in Tris buffer (50 mM Tris buffer, pH 8.0), and then 5 μg of Syb-2 was added to initiate the reaction. After 15 min the reaction was stopped by addition of SDS-PAGE loading buffer. Samples were heated for 10 min at 95 °C in SDS-PAGE loading buffer and loaded on 15% SDS-PAGE gel for electrophoresis and detection with Coomassie staining [28]. If the non-cleaved Syb-2 levels were greater than 75%, then the mAb was considered to have the most potent inhibitory activity “++”, between 50% and 75%, then medium potency “+”, or if less than 50%, it was considered to have no inhibitory activity “−”.

### 3.10. Determination of IC_50_ Values

The concentration leading to 50% inhibition (IC_50_) of selected scFvs or IgGs was measured using FRET for selected mAbs [39]. Briefly, 0.8 μM of YsCsY was first mixed with two-fold serially diluted scFv or IgG starting at a concentration of 50 nM. After pre-incubation for 15 min at 30 °C, 2 nM of BoNT/B LC was added. Emissions at 528 and 485 nm with a cutoff at 435 nm were measured at t = 0 and at 40 s intervals. The ratio 528/485 nm (R) was calculated to quantify YsCsY cleavage. The rate of change of R was considered as the cleavage rate of YsCsY. The initial reaction rate (*v*_0_) was then calculated by fitting the R of the first 400 s to a linear regression model: *Y* = *v*_0_*X* + C. Finally, the IC_50_ values were determined by fitting *v*_0_ and log mAb concentration to the sigmoidal dose-response (variable slope) model (GraphPad Prism, version 6.0, La Jolla, CA, USA). The use of 2 nM BoNT/B LC may have limited the ability to quantitate mAbs with IC_50_ below this level.

### 3.11. Random Mutation Library Construction and Sorting

A BoNT/B LC fragment library with random mutations was prepared using error-prone PCR with the primers pYD-For/pYD-Rev and DNA polymerase Paq5000 (Agilent Tech, Palo Alto, CA, USA) plus 12.5 μM MnCl_2_, as previously described [30]. The PCR product was then gel purified, inserted into the NcoI/NotI sites of the plasmid pYD4, and transformed into EBY100. The library was cultured in SD-CAA medium for 48 h, and then 50 mL of the culture was induced in 500 mL SG-CAA medium at 18 °C for 48 h. To determine the critical amino acid for mAb binding, taking mAb 1B10.1 as an example, the library was sorted by incubating the clones with 1B10.1 labeled with Alexa-647 and a mAb that binds a non-overlapping epitope; for example, mAb 2B23, labeled with Alexa-488. After labeling, the yeast population binding both mAbs was collected for the second round of sorting, in which the library was again incubated with 1B10.1-Alexa-647 and 2B23-Alexa-488. The population binding mAb 2B23 but not binding 1B10.1 was collected, grown, induced with SG-CAA and a third round of sorting using the conditions described above was performed. The sort output was plated and individual colonies displaying the BoNT/B LC analyzed for loss of binding to mAb 1B10.1. Clones with loss of binding were sequenced to identify mutations responsible for loss of binding. This process was repeated to identify the residues associated with loss of binding for the other mAbs.

### 3.12. Site-Directed Mutagenesis

Alanine mutants of BoNT/B LC were prepared following the manufacturers instructions for the QuikChange II-E Site-Directed Mutagenesis Kit (Agilent Tech, Palo Alto, CA, USA). Briefly, primers containing the mutation were used for PCR amplification with the plasmid pYD4 containing the BoNT/B LC gene (pYD4-BLC) or containing the BoNT/B LC-H_N_ gene (pYD4-B LC-H_N_) as a template for 18 cycles. The PCR product was digested by DpnI to remove the parental methylated and hemimethylated DNA, which was then purified by StrataClean Resin and transformed into *E. coli* XL1-Blue. The alanine mutants of BoNT/B LC or BoNT/B LC-H_N_ in pYD4 were than individually transformed into EBY100, grown in SD-CAA medium and induced for expression on the surface of EBY100. DNA sequencing was used to verify each construct.

### 3.13. Fine Epitope Mapping of mAb 1B10.1

The fine epitope map of mAb 1B10.1was determined by calculating the change of Gibbs free energy (ΔΔG) upon alanine-scanning mutagenesis of BoNT-LC-H_N_ as described [30]. Residues flanking those shown to knockout 1B10.1 binding were mutated to alanine by site-directed mutagenesis and displayed on the yeast surface. The K_D_ of 1B10.1 to the yeast-displayed for wild type and BoNT-LC-H_N_ mutants was determined by serial dilution of 1B10.1 F(ab). All K_D_ values were determined in triplicate. ΔΔG was calculated to evaluate the binding contribution of each amino acid in the epitope using the following formula:

ΔΔG (kcal/mol) = RT × ln(K_D_ − Mut/K_D_ − Wt)/1000

where, R = 1.9858775 cal/k∙mol; T (K). Using the ΔΔG value, the epitope was modeled on the surface of the crystal structure of BoNT/B (PDB ID: 1S0F [31]). Amino acids in the epitope were colored from red (the greatest ΔΔG) to wheat (the smallest ΔΔG) to show the different amino acid contributions to the epitope.

## 4. Conclusions

This work identifies for the first time mAbs that potently inhibit substrate cleavage by BoNT/B and defines their epitopes within the LC domain. The epitopes of the mAbs showing the greatest inhibition of BoNT/B activity mapped differently from the mAbs inhibiting substrate cleavage by BoNT/A, consistent with differences in how the cognate substrates bind the two BoNT serotypes. The mAbs presented here could form the basis of a therapeutic inhibitory cargo for reversal of paralysis due to BoNT intoxication via a delivery vehicle [26]. For example, liposomes containing inhibitory peptides and delivered into cells inhibited the neuroparalytic effect of BoNT/A *in vitro* [27]. Similar delivery vehicles could be used to deliver either the scFv mAbs or their genes to achieve reversal of paralysis. Inhibitory epitopes defined through this study could potentially be targeted for development of small molecule inhibitors. 

## Abbreviations

AAALAC/I: Assessment and Accreditation of Laboratory Animal Care, International
BoNTs: Botulinum neurotoxins
mAb: Monoclonal antibody
HC: Heavy chain
LC: Light chain
H_C_: BoNT binding domain
H_N_: BoNT translocation domain
K_D_: Equilibrium binding constant
CHO: Chinese hamster ovary
IgG: Immunoglobulin G
Fab: Antigen binding fragment of immunoglobulin with variable domain and first constant domain
scFv: Single-chain variable fragment
FACS: Fluorescent activated cell sorting
sb2: Synaptobrevin-2
SNAP 25: Synaptosomal-associated protein, 25kDa
Vh: Heavy chain variable region
Vk: Kappa chain variable region
Wt: wild type
IMAC: Immobilized metal affinity chromatography
PCR: polymerase chain reaction
PBS: Phosphate buffered saline
SD-CAA: Selective growth dextrose casamino acids media
SG-CAA: Selective growth galactose casamino acids media
SDS-PAGE: SDS-polyacrylamide gel electrophoresis
MFI: Mean fluorescence intensity
FRET: Fluorescence Resonance Energy Transfer
YsCsY: Yellow fluorescent protein (YFP)-synaptobevin-2-cyan fluorescent protein (CFP)-synaptobevin-2-YFP
PE: Phycoerythrin
IC_50_: the half maximal inhibitory concentration
ΔΔG: Change of Gibbs free energy
